# Temporal-order judgment of visual and auditory stimuli: modulations in situations with and without stimulus discrimination

**DOI:** 10.3389/fnint.2012.00063

**Published:** 2012-08-25

**Authors:** Elisabeth Hendrich, Tilo Strobach, Martin Buss, Hermann J. Müller, Torsten Schubert

**Affiliations:** ^1^Department of Psychology, Ludwig-Maximilians-Universität MünchenMunich, Germany; ^2^Department of Psychology, Humboldt-Universität zu BerlinBerlin, Germany; ^3^Institute of Automatic Control Engineering, Technische Universität MünchenMunich, Germany

**Keywords:** dual task, choice RT task, temporal order judgments, comparison, time of temporal order decision

## Abstract

Temporal-order judgment (TOJ) tasks are an important paradigm to investigate processing times of information in different modalities. There are a lot of studies on how temporal order decisions can be influenced by stimuli characteristics. However, so far it has not been investigated whether the addition of a choice reaction time (RT) task has an influence on TOJ. Moreover, it is not known when during processing the decision about the temporal order of two stimuli is made. We investigated the first of these two questions by comparing a regular TOJ task with a dual task (DT). In both tasks, we manipulated different processing stages to investigate whether the manipulations have an influence on TOJ and to determine thereby the time of processing at which the decision about temporal order is made. The results show that the addition of a choice RT task does have an influence on the TOJ, but the influence seems to be linked to the kind of manipulation of the processing stages that is used. The results of the manipulations indicate that the temporal order decision in the DT paradigm is made after perceptual processing of the stimuli.

## Introduction

To form an adequate representation of the environment, we often have to integrate visual and auditory information into a multisensory representation (Stein and Meredith, 1994; King, 2005; Spence, 2007). An important factor influencing this integration is the different processing duration of visual and auditory perceptual processing. Physically, an auditory signal coming from a certain source is slower in reaching the observer than a corresponding visual signal (Sugita and Suzuki, 2003). This is somehow compensated for by faster sound transduction than light transduction and by faster neural processing of auditory information in the human neural system (King, 2005).

Researchers have developed a number of paradigms that are directed at investigating the principles of information processing in different information modalities (i.e., perceptual latencies). Temporal-order judgment (TOJ) is one of these paradigms and it is commonly used for comparing perceptual latencies in different information modalities (e.g., Spence et al., 2001; Miller and Schwarz, 2006; Cardoso-Leite et al., 2007; Shi et al., 2008; Boenke et al., 2009). In a typical TOJ task, two stimuli are presented with varying stimulus onset asynchronies (SOAs) and participants are asked to indicate the temporal order of the two stimuli. The point in time at which the two stimuli are rated as presented at the same time, is called the Point of Subjective Simultaneity (PSS). Several factors have been identified to have an effect on the perception of temporal order. One of them is the modality of the stimuli (e.g., Hirsh and Sherrick, 1961; Rutschmann and Link, 1964; Roufs, 1974; Jaśkowski et al., 1990; Spence et al., 2001). Many authors consent that an auditory stimulus has to be presented after a visual stimulus to be perceived as simultaneous in a TOJ task (Hirsh and Sherrick, 1961; Dinnerstein and Zlotogura, 1968; Jaśkowski et al., 1990; Zampini et al., 2003; Keetels and Vroomen, 2005; Van Eijk et al., 2008; Boenke et al., 2009; but see, e.g., Rutschmann and Link, 1964). This effect is ascribed to the faster sound transduction in the human ear than light transduction in the human eye (King, 2005; Arrighi et al., 2006). Additionally neural transmission times are shorter in the auditory system than in the visual system (King, 2005). Therefore, the onset of the auditory stimulus has to be delayed compared to the onset of a visual stimulus if both ought to be perceived as simultaneous. Times of reported auditory delay vary from 5 ms (Hirsh and Sherrick, 1961) to 71 ms (Dinnerstein and Zlotogura, 1968). However, there are some studies that report the opposite effect of visual delays (e.g., Rutschmann and Link, 1964). According to Boenke et al. (2009), this might be explained by higher intensity of the visual stimuli and/or lower intensities of the auditory stimuli. Stimulus intensity is therefore another factor that seems to influence TOJ (Neumann, 1982; Boenke et al., 2009).

However, what has, to our knowledge, not been studied so far, is whether the particular processing requirements that are associated with the auditory and the visual stimulus affect the TOJs in addition to temporal delay and intensity characteristics. A number of studies have advocated for a close relation between perception and action planning (e.g., Deubel and Schneider, 1996; Deubel et al., 1998; Witt et al., 2005; Zwickel et al., 2007; Witt and Proffitt, 2008), pointing to mutual dependencies between processes involved in the early perception of sensory information and in processes planning actions with the perceived sensory information. Witt et al. (2005), for instance, found that people perceive a target that is just beyond arm's reach as closer when they intend to reach it with a tool compared to when they plan to reach it without the tool. Deubel and colleagues also showed the close connection between intended actions and perceptual processing. For instance, Deubel and Schneider (1996) showed that the planning of an action has an early influence on perceptual processes. In a dual-task (DT) paradigm, they asked participants to plan a saccade to a specific location. Additionally, the participants had to discriminate between the symbols “E” and “mirror-E,” either at the target location of the saccade or at an adjacent location. Stimulus discrimination performance was best, if the two tasks, i.e., planning a saccade and discriminating, involved the same stimulus at one location and dropped if they involved different stimuli at different locations. Deubel et al. (1998) showed similar findings for the planning of a manual reaching task. If action planning requirements influence early perceptual processes, e.g., by influencing the allocation of attentional resources to the processed stimuli, then this may have an additional effect on the processing speed of the perceptual stimuli. It is well known that attention facilitates the detection (e.g., Posner, 1980) and the identification of visual stimuli (e.g., Desimone and Duncan, 1995) and this may influence subsequent judgments about stimulus features, e.g., the temporal order of their presentation.

A way to investigate this research question is to add the requirement to carry out a discriminative response on the processed visual and auditory stimuli in the context of a TOJ task. This can be done by administering different types of visual and auditory stimuli, which have to be discriminated in order to perform a choice reaction in addition to the TOJ. The question of TOJ under the condition of an additional choice requirement is of relevance because visual and auditory information often not only have to be noticed but also require an appropriate reaction from the observer in a laboratory context as well as in a real world environment. In this situation it is of special interest, whether such an enforced choice reaction will or will not have an effect on the order with which the auditory and the visual signal are perceived. From a theoretical perspective, an answer on this question may also provide valuable insights into our understanding of the processing architectures of TOJ tasks and DT situations with variable task orders. It should be mentioned at this point, that simultaneity judgments (SJ) are an alternative method to investigate time characteristics of stimulus processing and this method can show results different from TOJ results (see Van Eijk et al., 2008). For SJs, participants are asked to indicate whether two stimuli are present at the same time or not. We choose TOJ instead of SJs, because the determination of temporal order of stimuli is more complex and therefore more sensitive to detect possible differences between different conditions within TOJ (e.g., high or low stimulus contrast) and between TOJ and DT. Compared to that, indicating whether two stimuli are presented at the same time or not, is relatively easy. Therefore, we considered TOJs to be the more appropriate paradigm to investigate timing of stimulus processing in contrast to SJs.

Several authors conducted experiments on DT with variable intervals between stimuli (SOA) and unpredictable task order, which necessitated an additional judgment of the temporal order of the two stimuli (e.g., De Jong, 1995; Luria and Meiran, 2003; Sigman and Dehaene, 2006; Szameitat et al., 2006). In these DT situations participants are presented with stimuli in different modalities and they have to perform a choice reaction on the stimuli. Most often participants are required to perform the two tasks in the order of the stimulus presentation. While this task basically requires first TOJ about the presentation of stimuli in different modalities, it subsequently requires that different visual and auditory stimuli need to be distinguished, related to a pre-specified response category and a subsequent motor response needs to be selected and executed (Umiltà et al., 1992; De Jong, 1995; Sigman and Dehaene, 2006).

Surprisingly, while TOJs play a role in research on DT situations with unpredictable SOA, only one study, i.e., De Jong (1995), compared the response order results of DTs to the response order in typical TOJ tasks. In particular, De Jong realized this comparison when he used TOJ as a control condition for a DT experiment with varying task order. In the DT task, an auditory and a visual choice RT task were presented and participants were either asked to respond in the order the two stimuli were presented (forced-order) or they received no specific instruction regarding response order (free-order). To certify that participants were able to judge the order of the two stimuli correctly, De Jong added a control condition in which the participants were exclusively asked to judge the order of the stimulus presentation without conducting discriminative choice reactions. The results of this study showed that, in a substantial number of trials, the participants responded to the stimuli in the opposite order of their presentation (“response reversals”) in both instruction-conditions. In the control condition, in which participants judged only the order of the stimulus presentation, the number of response reversals was much lower than in the experimental condition. A reason for the higher number of response reversals in the experimental condition (DT) compared to the number of response reversals in the control condition (TOJ-type) could be the additional requirement to make speeded responses to the stimuli.

However, De Jong (1995) discarded this idea of an impact of the additional speeded responses on temporal order decision as unlikely and did not pursue it any further. This decision was based on findings from a study of Sternberg et al. (1971), who combined a TOJ task with an RT task. According to De Jong, the results reported by Sternberg et al. suggested that “such interfering effects (caused by an additional RT task) were probably quite minimal” (p. 15). However, the tasks used by Sternberg et al. were quite different from the ones De Jong had used. Sternberg et al. had presented an auditory and a cutaneous stimulus. A cue before one of the two stimuli told the participants that they had to pull a lever as quickly as possible in reaction to the cued stimulus and after that judge the order of the two stimuli by pressing a corresponding button. Thus, participants had to do first a reaction-time task to one of the stimuli and then, in a second step, judge the order of the stimuli. In the study of De Jong, however, participants had to do two choice reaction tasks plus a TOJ and respond to the stimuli in the order of their presentation.

In our opinion, the two studies, i.e., De Jong (1995) and Sternberg et al. (1971), focused on two task conditions, which exposed highly different task demands for the participants and their results are not comparable with each other. Therefore, we think that the question whether an additional choice-RT task on the presented stimuli has an influence on TOJs has not yet been addressed sufficiently and, aim to shed new light on it. Based on De Jong's results (De Jong, 1995), we expect that an additional identification task has an influence on TOJs. The study of De Jong, however, did not provide any details about the specific processing architecture of TOJ with and without selecting responses following stimulus identifications.

A second aim of this study was to investigate, when, precisely, the judgment of temporal order takes place during task processing in pure TOJ tasks and in DT situations with unpredictable task order. As already noted, task processing in a RT task is assumed to consist of several consecutive processes or stages (Sternberg, 1969; Sanders, 1980, 1990): perception, response selection, and response execution. At some point in time during the information processing chains of two tasks the temporal order of the two stimuli has to be decided. First empirical hints about the location of the order decision in DT situations come from Sigman and Dehaene (2006). These authors investigated whether the prolongation of certain processing stages has an influence on the processing order of the two component tasks in a DT situation. To analyze this question, they used a DT paradigm with unpredictable stimulus order, in which participants were asked to respond to an auditory and a visual stimulus in the order of their presentation. Sigman and Dehaene found that the prolongation of the perception stage of the visual task (if it was presented first) led to an increase in response reversals, while the prolongation of the response-selection stage of the visual task did not. To explain their results, they assumed that task order is decided after the perceptual processes of the first presented stimulus, in a definite executive control processing stage (see also Lien et al., 2003; Liepelt et al., 2011; Strobach et al., in press for alternative ideas on such control processes).

To investigate the precise location of the task order decision in the TOJ paradigm and in the DT paradigm, we manipulated the length of the first two task processing stages (perception and response-selection stage) in the DT paradigm and the first stage, the perception stage, in the TOJ paradigm. We tried to do so in a more direct way, in comparison to Sigman and Dehaene (2006), and compared the effects on response order in DT with effects in TOJ where feasible, as there is no response-selection stage in a TOJ task which can be manipulated. For the particular case of task order decision in DT situations, the manipulations could lead to three different outcomes: if the TOJ occurs at the very beginning of task processing, then both manipulations, the manipulation of the perception stage and the manipulation of the response-selection stage (resulting in manipulations of the stage latencies), should fail to show an effect on response order. This is because manipulations of perceptual and response-selection latencies do not affect the outcome of a TOJ process located before these manipulated stages. If the order judgment happens after perception, but before the response-selection stage, then only the manipulation of the perception stage should have an influence on response order. This hypothesis results from the assumption that manipulations of perceptual stage latencies but not latencies of response selections have an effect on the outcome of a TOJ process located between the former and the latter manipulated stage. If the judgment about the temporal order of the two stimuli occurs after the response-selection stage, then both manipulations have an effect on response order. This is because we assume that manipulations of perceptual and response-selection latencies affect the outcome of a TOJ process located after these manipulated stages.

For the particular case of order decision in the TOJ paradigm, the manipulation of the perception stage is decisive: if this manipulation provides an effect on the order decision, then the decision is located after the perception stage.

In sum, the first aim of this study was to investigate whether TOJ is principally affected by the requirement to perform a discriminative response on the perceived stimuli in addition to the TOJ. Furthermore, we investigated the point in time at which the decision about the temporal order of the two stimuli is made in the condition with additional identification of the stimulus. For that purpose, we manipulated the length of the perception stage (Experiment 1) and of the response-selection stage (Experiment 2) of one of the two tasks.

## Experiment 1: manipulation of the perception stage

In Experiment 1, we compared “pure” TOJ in a TOJ paradigm with order judgments in a DT paradigm requiring the additional identification of the stimuli plus a response selection in the component tasks. We presented an auditory and a visual task in varying order. For the visual task, one of three numbers was shown randomly. For the auditory task, one of three different tones was presented. Usually, in the TOJ paradigm participants have to press one of two keys indicating whether stimulus A or stimulus B was presented first. In order to minimize differences between the two tasks, participants in the TOJ-task condition judged the order of the two stimuli by pressing two buttons in the corresponding order of stimulus presentation. In the DT condition, participants had to identify the stimuli and press two corresponding buttons (1 out of 3 for the visual task and 1 out of 3 for the auditory task) in the order of the stimulus presentation. The length of the perception stage of the visual task was manipulated by weak or strong contrast of the presented numbers and their background.

### Method

#### Participants

Eighteen participants (12 female) took part in the experiment. The participants were all students of the LMU Munich, who received course credit or payment (8 Euro/hour) for their participation. The average age was 25.0 years (SD = 3.0 years). All subjects were right-handed and reported normal or corrected-to-normal vision and hearing.

#### Apparatus and stimuli

The participants were tested individually in a sound-attenuated, darkened room. They sat in front of a CRT monitor (85 Hz) at a distance of about 60 cm and wore headphones. Responses were given on the QWERT-keyboard. The experimental code was written in Presentation (Version 14.4 02.24.10) and run on a Dell Optiplex GX620 with Windows XP Professional.

Three digits were presented as the visual stimuli: “2,” “5,” or “9.” The numbers were either presented with strong contrast (white font color, 55 cd/m^2^, against dark-gray background) or with weak contrast (gray font color, 0.09 cd/m^2^, against dark-gray background). Each number subtended a visual angle of 1° × 1.5° (1 cm × 1.5 cm). Dark gray background (0.11 cd/m^2^) was used instead of black background to minimize visual after-effects. The auditory stimuli were three sine-wave tones with frequencies of 250, 500, and 1000 Hz and a volume of 58 decibel. They were presented via headphones. Both types of stimuli, visual, and auditory, were presented for 200 ms each.

#### Design and procedure

The experimental design formed a 2 × 2 × 7 factorial model with task condition (TOJ vs. DT), contrast of the visual stimulus (weak vs. strong contrast) and SOA (−400 ms, −120 ms, −60 ms, 0 ms, +60 ms, +120 ms, +400 ms) as within-subjects factors.

In the TOJ-task condition, participants completed 20 practice trials before starting with the main experiment. Each trial started with the presentation of a fixation point in the centre of the screen for 500 ms. The fixation point was followed by a blank screen for 600 ms, then the first stimulus presentation (i.e., either visual or auditory) and, after a variable SOA, the second presentation (i.e., auditory or visual, respectively). Participants then responded by pressing the “c”-key to the auditory stimulus and the “,”-key to the visual stimulus in the order of the perceived stimulus presentation order. Each trial had a constant length of 4,500 ms.

In the DT condition, participants completed two practice blocks for the single tasks (15 trials each) and one practice block with both tasks (20 trials). The procedure of trials with both tasks was identical to the TOJ-task condition. In contrast to the TOJ-task condition, however, participants responded by pressing “y,” “x,” or “c”-key for low, middle, and high tone (i.e., auditory task) and “,”; “.”; or “-”-key for 2, 5, and 9 (i.e., visual task) in the DT-part, respectively. Feedback on the correctness of the responses was given in the practice blocks.

All possible combinations of SOA (±400 ms, ±120 ms, ±60 ms, 0 ms), visual stimuli (2, 5, 9), and auditory stimuli (250, 500, 1000 Hz) resulted in 63 different trial types. The order of these trial types varied randomly for each participant in each block. For each task condition (TOJ and DT), 3 blocks with strong-contrast and 3 blocks with weak-contrast visual stimuli were presented in alternating order; therefore, 378 trials were presented for each task condition. Half of the participants started with a strong-contrast block, the other half with a weak-contrast block. Response order and PSS were measured as dependent variables.

The complete experimental session lasted approximately 90 min. It consisted of two parts, TOJ and DT, presented in this order for every participant. Participants had the opportunity to have a short break between the two parts.

### Results

#### Temporal-order judgment

To compare the TOJs between the TOJ-task condition and the DT condition, we calculated the percentage of trials in which the tone was reported as first for each task condition, contrast condition, and SOA. The percentage of trials in which the tone was reported as first was submitted to an analysis of variance (ANOVA) including the within-subjects factors task condition (TOJ vs. DT), contrast (strong vs. weak contrast), and SOA (±400 ms, ±120 ms, ±60 ms, 0 ms).

**Figure 1:**
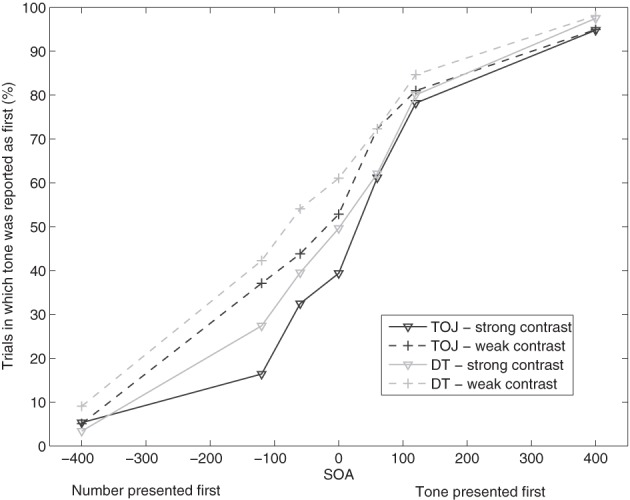
**Proportion of trials in which the participants reported the tone task as first in the conditions of temporal-order judgment (TOJ) and dual task (DT) in Experiment 1, each with two contrast conditions (strong and weak contrast)**.

As illustrated in Figure 1, participants tried to follow the instruction to respond in the order of presentation, which is reflected in a significant effect of SOA, *F*_(6, 102)_ = 145.119, *p* < 0.01. As can be seen in Figure 1, participants had a higher number of tone-first responses under conditions with positive SOA, i.e., conditions in which the tone was presented before the visual stimulus compared to conditions with negative SOA, i.e., conditions in which the tone was presented second.

We also found a significant difference between the task conditions, *F*_(1, 17)_ = 6.029, *p* < 0.05. Participants responded to the auditory task first significantly more often in the DT condition (*m* = 55.8%) than in the TOJ-task condition (*m* = 51.1%). The ANOVA also revealed a significant interaction of task condition and SOA, *F*_(6, 102)_ = 2.276, *p* < 0.05. Further *t*-tests revealed significant differences between the two task conditions at SOAs −120 ms [*t*_(17)_ = −2.184, *p* < 0.05], −60 ms [*t*_(17)_ = −2.130, *p* < 0.05], and 0 ms [*t*_(17)_ = −2.613, *p* < 0.05]. In the DT condition, participants reported the tone task as first more often than in the TOJ-task condition.

The manipulation of the contrast led to a significant effect, *F*_(1, 17)_ = 22.962, *p* < 0.01. Participants reported the tone as first more often in the condition with weak contrast (*m* = 57.8%) than in the strong-contrast condition (*m* = 49.1%). There was also a significant interaction of contrast and SOA, *F*_(6, 102)_ = 7.052, *p* < 0.01. The percentage of trials in which the participants reported the tone task as first was higher for weak contrast at the SOA levels −400 ms [*t*_(17)_ = −2.536, *p* < 0.05], −120 ms [*t*_(17)_ = −4.367, *p* < 0.01], −60 ms [*t*_(17)_ = −4.473, *p* < 0.01], 0 ms [*t*_(17)_ = −3.226, *p* < 0.01], and 60 ms [*t*_(17)_ = −3.578, *p* < 0.01]. There were no further interactions.

#### Point of subjective simultaneity (PSS)

**Figure 2:**
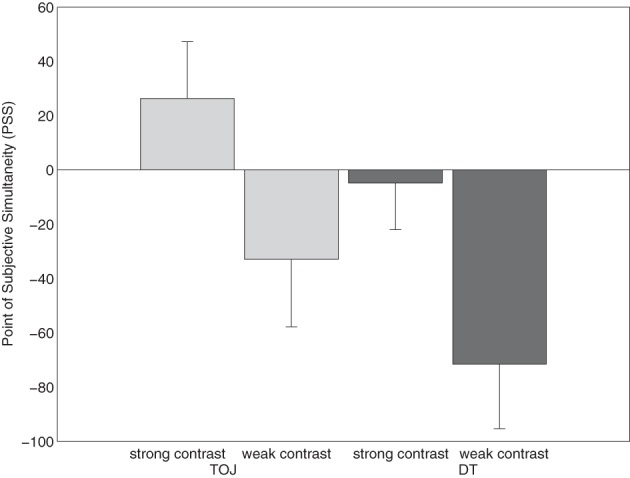
**Points of subjective simultaneity (PSS) for the two task conditions temporal-order judgment (TOJ) and dual task (DT) in Experiment 1, each measured in two contrast conditions, strong and weak contrast**.

The PSS denotes the SOA, at which the participants report the tone as first in 50% of the trials. It was calculated by fitting logistic regression functions to the data of each participant. For each condition, the PSS was calculated by estimating the 50% performance point on the fitted logistic function (Treutwein and Strasburger, 1999). As illustrated in Figure 2, for the TOJ-task condition, the mean PSSs were 26.3 ms for the strong-contrast condition and −33.0 ms for the weak-contrast condition. The results indicate that, in the strong-contrast condition, the number digit had to be presented 26 ms after the tone to be perceived as simultaneous. In the weak-contrast condition, the number had to be presented 33 ms before the tone task to be perceived as simultaneous. In the DT condition, the mean PSSs were −4.8 ms for the strong contrast condition and −71.5 ms for the weak contrast condition. In both contrast conditions, the number had to be presented before the tone to be perceived as simultaneous. PSSs were submitted to a 2 × 2 ANOVA with task condition (TOJ vs. DT) and contrast (strong vs. weak contrast) as within-subjects-factors. The ANOVA revealed a significant effect of task condition, *F*_(1, 17)_ = 4.451, *p* < 0.05. The PSS was significantly more negative in the DT condition (*m* = −38.2 ms) than in the TOJ-task condition (*m* = −3.4 ms). The factor contrast did also show a significant effect, *F*_(1, 17)_ = 19.878, *p* < 0.01. PSS values were significantly more negative in the condition with weak contrast (*m* = −52.2 ms) than in the strong-contrast condition (*m* = 10.7 ms). The interaction of task condition and contrast was not significant, *F*_(1, 17)_ < 1.

### Discussion

In a TOJ-task, participants have to indicate the order of two stimuli which are presented with varying SOAs. In Experiment 1 we investigated whether the requirement to identify and discriminate the stimuli plus subsequent response selection processes in an auditory-visual DT have an effect on the TOJ. Additionally, the perception stage of the visual task was manipulated to localize the processing stage at which the decision about the temporal order is made. In fact, the present contrast manipulation aims at testing whether the judgment is made before or after perceptual processes associated with the contrast manipulation.

The results of Experiment 1 show that TOJ, as measured by the response order, is influenced by the kind of task requirement the participants have to complete. When participants have to judge the temporal order of two stimuli and, additionally, have to identify the stimuli, then they report the auditory stimulus significantly more often as first compared to when they do not have to identify the specific auditory stimulus. This effect was especially pronounced for trials in which the visual stimulus was presented first. A possible reason for this observation might be that the perceptual stage of the visual task was manipulated in Experiment 1. Participants might have considered the visual task to be the more difficult one and therefore might have preferred to do the easier auditory task first. We will return to this effect in Experiment 2 and in the General discussion.

Furthermore, we found an effect of task condition on the PSS, which fits to the results of the TOJ data.

For the issue of the localization of the point in time at which the temporal order decision is made, the results of the manipulation of the visual perception stage are important. The results showed that the contrast manipulation of the visual stimulus had an effect in both task conditions, the TOJ- and DT condition. In the condition with weak contrast, the visual task had to be presented earlier than in the condition with strong contrast to be perceived as simultaneous. Importantly, concerning the point in time at which the TOJ occurs in the DT- and TOJ-task condition, we assume that it must happen after the perception stage, because the manipulation of the perception stage influences the TOJs in both the TOJ- and the DT condition.

This effect was especially pronounced for trials in which the visual task was presented first. These results are in line with Boenke et al. (2009), who argued that relative stimulus intensity influences the PSS in TOJ-tasks. More specifically, they claimed that studies which found that the visual stimulus has to be presented before the auditory stimulus to be perceived as simultaneous used auditory stimuli of higher intensity and/or visual stimuli of lower intensity than studies who found the opposite. This indicates that higher intensity of the visual stimulus leads to a shift of the PSS. The results of the TOJ-task condition in Experiment 1 support this idea. In the condition with strong contrast of the visual stimulus, the PSS is positive, which means that the auditory stimulus has to be presented before the visual stimulus to be perceived as simultaneous. In the condition with low visual intensity, however, the PSS is negative; thus, there is a requirement to present this visual before the auditory stimulus to generate a percept of simultaneous stimulus presentation.

In the DT condition, we also found a shift of the PSS from strong to weak contrast. The PSS in the condition with low stimulus intensity was more negative than the PSS in the condition with high stimulus intensity. This means, in the condition with low intensity the visual stimulus has to be presented even longer before the auditory stimulus than in the high intensity condition to be perceived as simultaneous.

## Experiment 2: manipulation of the response-selection stage

The results of Experiment 1 showed that the manipulation of the perception stage has an influence on the judgment of temporal order. Thus, the judgment must be localized after the perception stage because otherwise we should not have found an effect of the duration of the visual stimulus on judgment order. In Experiment 2, we aimed to further specify the temporal location of the order judgments within the particular task processing architecture of the current task situations. While the processing chain in TOJ tasks is mostly restricted to the perception of the stimuli, the comparison of their presentation times, and the programming of motor responses, the component tasks in the DT situation involve an additional response-selection stage each. In Experiment 2, we aimed to assess whether the TOJ in the DT situation is located before the response-selection stage or not. Note that the findings of Experiment 1 leave open that question because they localized the order judgment only non-specifically as later than the perception stage. For that purpose, in Experiment 2 we manipulated the duration of the response-selection stage in the visual task of the DT situation by manipulating the stimulus-response compatibility.

If judging the temporal order occurs after the response-selection stage, then the manipulation of its duration should have a notable effect on the order judgments. In case judging the order occurs before that stage, it should not have an effect on order judgments. We manipulated the stimulus-response compatibility of the visual task by administering a compatible condition, in which the numbers (2, 5, 9) presented in the visual task were mapped to keys of right hand motor responses according to numerical magnitude. In the incompatible condition, numbers were mapped in a non-standard way to the response keys of the right hand, the 5 to the leftmost key, the 9 to the middle key, and the 2 to the rightmost key. This manipulation should affect merely the duration of the response-selection stage in the visual task of the DT condition (Sanders, 1980; McCann and Johnston, 1992).

### Method

#### Participants

In Experiment 2, 19 (17 female) participants took part, who had not participated before. The participants were again students of the LMU, who received course credit or payment (8 Euro/hour) for their participation. The average age was 23.9 years (SD = 3.6 years). All subjects except for one were right-handed and all reported normal or corrected-to-normal vision and hearing.

#### Apparatus, stimuli, design, and procedure

These characteristics of Experiment 2 were identical to Experiment 1 with the following exceptions. In Experiment 2, the stimulus-response mapping of the visual task was manipulated. The mapping could be either compatible or incompatible. In the compatible-mapping condition, the participants responded to the three numbers 2, 5, and 9 by pressing the “,”; “.”; and “-”-key, respectively. In the incompatible-mapping condition, the numbers were mapped to the same keys, but in a different, non-standard order: 5 was mapped on the “,”-key, 9 on the “.”-key, and 2 on the “-”-key.

The design formed an incomplete factorial model. As there was only one response key for all three different stimuli in the TOJ-task condition, the stimulus-response mapping could only be manipulated for the DT condition. For the TOJ-task condition, participants completed three blocks of TOJs after the completion of 20 practice trials. For the DT condition, three blocks with compatible stimulus-response mapping and three blocks with incompatible stimulus-response mapping of the visual stimuli were presented in alternating order. Half of the participants started with a block with compatible mapping, the other half with an incompatible-mapping block. Before the six experimental DT-blocks, participants completed three practice blocks for the single tasks (20 trials each for auditory task, visual task with compatible stimulus-response mapping and visual task with incompatible stimulus-response mapping) and two practice block with both tasks with the two different stimulus-response mappings (30 trials each). The complete experimental session lasted approximately 75 min and consisted of TOJ and DT, which were conducted in this order by every participant.

### Results

#### Temporal-order judgments

**Figure 3:**
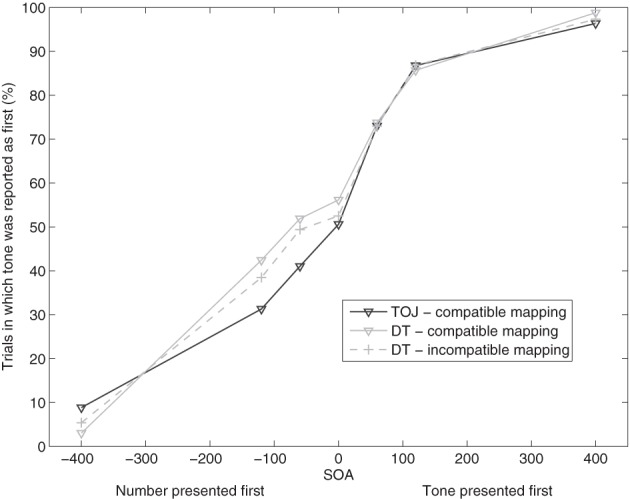
**Proportion of trials in which the auditory task was reported as first in the conditions of temporal-order judgment (TOJ) and dual task (DT) in Experiment 2.** Two DT conditions were presented: DT with compatible stimulus-response mapping and DT with incompatible stimulus-response mapping.

We calculated the percentage of trials in which the participants reported the auditory stimulus as first for every task condition and every SOA and present them in Figure 3. The data were submitted to a repeated-measures ANOVA with task condition (TOJ, DT compatible, and DT incompatible) and SOA as within-subjects factors.

The factor SOA was significant, indicating that the percentage of trials in which the auditory task was responded to first, increased with increasing SOA, *F*_(6, 108)_ = 104.992, *p* < 0.01. Thus, similar to Experiment 1, participants generally tried to follow the instructions to judge the order of the stimuli because as can be seen in Figure 3, the proportion of trials in which the auditory stimulus was reported as first, was higher at positive SOAs compared to negative SOAs.

We did not find a significant effect of the factor task condition, *F*_(1.195, 21.510)_ < 1, (Greenhouse-Geisser corrected) on order judgment. Because that factor reflects the difficulty manipulation in the DT condition, the lacking effect of task condition reflects the fact that response order did not differ significantly between the parts TOJ, DT compatible, and DT incompatible. The manipulation of the stimulus-response mapping in the DT condition had no effect on the TOJs consistent with the assumption that the temporal-order decision occurred before the response-selection stage in the DT condition. Also, the interaction between SOA and task condition was not significant, *F*_(12, 216)_ = 1.543, *p* = 0.19. However, visual inspection of the data suggested a difference between the task conditions for the negative SOAs (trials in which the number was presented first). Indeed, if only TOJ and DT compatible data were included in the analysis, we found a significant interaction of task condition and SOA, *F*_(6, 108)_ = 2.343, *p* < 0.05. One-tailed *t*-tests showed significant differences between the task conditions for the SOAs −400 ms, −60 ms, and 400 ms. At SOA −400, the proportion of trials in which the participants reported the auditory task as first was higher in the TOJ-task condition than in the DT condition, *t*_(18)_ = −1.767, *p* < 0.05. At SOA −60, *t*_(18)_ = 1.868, *p* < 0.05, and SOA 400, *t*_(18)_ = 2.117, *p* < 0.05, the proportion of trials in which the participants reported the auditory task as first, was higher in the DT condition than in the TOJ-task condition. The results indicate a similar pattern as in Experiment 1, where the proportion of trials in which the tone was reported as first was significantly higher in the DT condition than in the TOJ condition, especially for negative SOAs, i.e., trials in which the number was presented first.

#### Point of subjective simultaneity (PSS)

**Figure 4:**
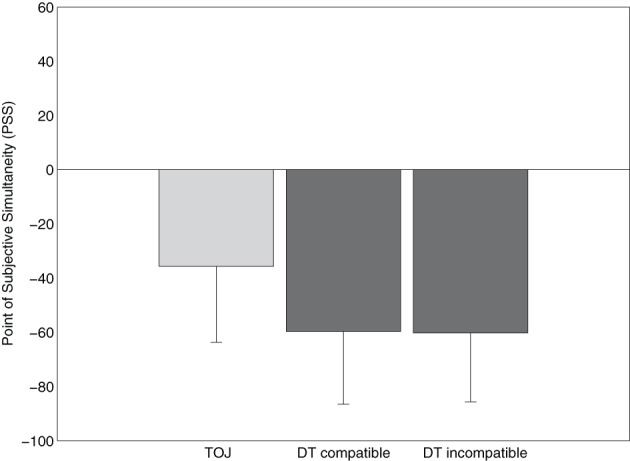
**Points of subjective simultaneity (PSS) for the temporal-order judgment task (TOJ) and the two dual-task (DT) conditions (compatible and incompatible stimulus-response mapping) in Experiment 2**.

Again, we calculated the PSSs by submitting the data of each subject to separate logistic regression analyses for each task and mapping condition. Then we calculated the mean PSS of all participants by estimating the 50% point of the logistic function, at which the participants report the auditory task and the visual task as first equally often (see Figure 4).

For the TOJ-task condition, the mean PSS amounted to −36 ms, which indicates that the tone had to be presented 36 ms after the number stimulus to be perceived as simultaneous. In the DT condition, the mean PSS amounted to −60 ms for both the conditions with compatible and incompatible response mappings. We submitted the PSS-values to a repeated-measures ANOVA with task condition as within-subjects factor (TOJ, DT compatible, and DT incompatible). This ANOVA revealed no effect of task condition, *F*_(2, 38)_ < 1, suggesting that the three different conditions, including the two DT conditions with different response selection difficulty, did not differ with respect to PSS.

### Discussion

The results of Experiment 2 show that the TOJ in the DT condition was not influenced by the manipulation of the response-selection stage. Neither was the PSS shifted by the manipulation when comparing across all conditions. These results indicate that the point in time at which the TOJ is made in the DT condition, was located before the response-selection stage, as the manipulation of this stage had no effect on TOJ.

An issue which needs additional consideration is the effect of the task condition on the overall order performance: in Experiment 2, the ANOVA did not reveal a main factor of task condition, which, on first glance, might be puzzling because in Experiment 1 we found that order judgments differed between the TOJ and the DT conditions. Thus, while we could not find an overall effect of task condition (including TOJ, DT compatible, and DT incompatible as levels) in Experiment 2, the visual inspection of the data suggested an effect of task condition for those trials in which the visual stimulus was presented first. This visual impression was confirmed when we included only the data of the TOJ and the DT compatible condition in the analysis. In that particular case, we obtained a significant interaction between task condition and SOA, which indicates a similar response pattern as in Experiment 1 thus replicating those findings. We will come back to this issue in the “General Discussion.”

In sum, the current findings suggest that the order judgments do not differ between the DT compatible and the DT incompatible conditions in Experiment 2, suggesting that the order decision must have taken place before the response-selection stage in the component tasks of the DT condition.

## General discussion

In the present experiments, one aim was to determine the point in processing when the decision about the temporal order of two presented stimuli is made. The other aim was to investigate whether the additional insertion of a response selection requirement for the processing of the stimuli in a TOJ context has an influence on TOJs.

In order to address the first aim, we manipulated the perception stage of the TOJ task and the DT in Experiment 1 and the response-selection stage of the DT in Experiment 2. As the manipulation of the perception stage had an effect on TOJs in both task conditions, we conclude that in both conditions the decision about the temporal order occurs after the perceptual stage. Experiment 2 showed that the manipulation of the response-selection stage did not have an influence on TOJ in the DT condition. For the DT condition, we therefore conclude that the decision about the temporal order of the two stimuli is made after perceptual processes and before the response-selection processes start. This finding corresponds with the findings of Sigman and Dehaene (2006), who also found that a manipulation of the perception stage does have an influence on response order, while a manipulation of the response-selection stage does not. The authors hypothesized that an executive process determines the order of the two stimuli which is located after the perceptual processes of the first task.

Concerning the question whether the additional insertion of a choice reaction has an influence on TOJs, the current data suggest that this is indeed the case. We found a difference between the TOJs in the TOJ-task and the DT conditions in Experiment 1. In the DT condition, the auditory task was reported as first more often than in the TOJ-task condition, especially in trials in which the visual task was presented first. In Experiment 2, we found a similar result, albeit not as clear as in Experiment 1. The auditory task was reported as first more often in the DT condition than in the TOJ-task condition. This effect appeared especially in those trials in which the visual task was presented first. Although the data in Experiment 2 did not show a general effect of task condition, the interaction between task condition and SOA indicates a similar pattern as in Experiment 1. The results of both experiments suggest that the additional requirement to discriminate between different stimuli in the DT condition has an influence on temporal order decisions. This effect might be the result of differences in attention allocation between the two task conditions, which result in differences in perception speed (e.g., Posner, 1980; Desimone and Duncan, 1995). Most importantly, we can thereby show, that in addition to the already known factors that have an influence on the perception of temporal order (e.g., stimulus modality, see e.g., Hirsh and Sherrick, 1961; Rutschmann and Link, 1964; Roufs, 1974; Jaśkowski et al., 1990; Spence et al., 2001), also specific task requirements influence temporal order decisions.

What are the relations between the current findings and findings of earlier saccadic studies (e.g., Deubel and Schneider, 1996)? As reported in the introduction, Deubel and Schneider (1996) showed in a DT study, that the planning of a saccade to a target stimulus improves perceptual processing of said visual stimulus in a concurrent discrimination task by allocating attention to it. Discrimination performance and thereby perception of the visual stimulus in this study is best when the saccade and the discrimination task involve the same location compared to when they involve two stimuli at different locations. Our results are also consistent with the assumption that visual stimulus processing (in relation to auditory stimulus processing) is potentially improved under particular conditions. That is, the visual task is reported as first more often in the TOJ-task condition requiring no response selections when contrasted to the DT condition that requires these selection processes in two tasks.

Why is the effect of task condition especially prominent in the trials in which the visual task was presented first? In Experiment 1, the perception stage of the visual task was manipulated in both task conditions, first in the TOJ-task condition followed by the manipulation in the DT condition. It could be that further task changes, like the addition of a discrimination requirement in the DT condition, had a stronger effect on the manipulated visual task. Compared to the TOJ condition, the processing of the visual task might have been slowed by this additional processing requirement because the stimulus processing rate is slowed per se or additional information may be processed when participants are instructed for stimulus discrimination. What might also play a role in explaining the prominent effect of task condition in trials in which the visual task was presented first is the usually faster processing of auditory stimuli compared to visual stimuli, which was particularly shown in TOJ studies (e.g., Hirsh and Sherrick, 1961; Dinnerstein and Zlotogura, 1968; Jaśkowski et al., 1990; Zampini et al., 2003; Keetels and Vroomen, 2005; Van Eijk et al., 2008; Boenke et al., 2009; but see e.g., Rutschmann and Link, 1964, for the opposite effect).

In Experiment 2, there was no manipulation of the TOJ-task condition, but still an interaction of task and SOA was found, when the TOJ-task condition was compared with the DT condition with compatible mapping. The same interaction was found in Experiment 1. In Experiment 2, it was also the visual task that was manipulated in the DT condition. Therefore, the same possible explanations as just mentioned for Experiment 1 might apply here: the additional processing stages necessary for DT compared to TOJ-tasks might have a greater effect on the visual task for the aforementioned reasons (see above).

A recent study by McDonald et al. (2005) found that an attended object is reported as being presented earlier than a simultaneously presented unattended object in a TOJ task. Attention was modulated by cueing one of the two visual stimuli with a sound. The authors recorded event-related brain potentials (ERPs) during the task and found that the attended stimulus was not processed faster than the unattended (which would have been indicated by latency shifts in early ERPs). Instead, attention had an effect on the amplitude of the ERPs: the attended stimulus showed a higher amplitude than the unattended one. The authors suggest that these attention-induced enhancements in signal strength of the cued stimulus are then “interpreted as a timing difference by a later comparator mechanism” (McDonald et al., 2005, p. 1201). In regard to our study, this could mean that the manipulation of the visual task led to a shift in attention to the auditory task, either because of an alerting quality of the auditory signal or because of differences in difficulty between auditory and visual task. This attention shift could have led to a strengthening of the auditory signal, which would have been interpreted as a difference in presentation time. In order to investigate this assumption, further experiments have to be done.

Summing up, our study gives new evidence for the time in processing at which the decision about the temporal order of two stimuli is made, both in a TOJ-task and a DT. Also, we could show that the additional task requirement to discriminate the stimuli has an influence on the TOJs of a visual and an auditory stimulus.

### Conflict of interest statement

The authors declare that the research was conducted in the absence of any commercial or financial relationships that could be construed as a potential conflict of interest.
